# Clinical characteristics and genetic features of 35 cases of adverse reactions to Bacillus Calmette-Guérin vaccine in children

**DOI:** 10.3389/fcimb.2025.1570382

**Published:** 2025-05-21

**Authors:** Houyu Chen, Xiaotao Yang, Yi Huang, Feng Jiao, Houxi Bai, Ying Zhu, Penghao Cui, Haifeng Jin, Yan Guo, Yanchun Wang, Yonghan Luo

**Affiliations:** ^1^ Second Department of Infectious Disease, Kunming Children’s Hospital, Kunming, Yunnan, China; ^2^ Faculty of Life Science and Technology, Kunming University of Science and Technology, Kunming, Yunnan, China; ^3^ Department of Reproductive Gynecology, The First People’s Hospital of Yunnan Province, Kunming, China

**Keywords:** Bacillus Calmette-Guérin, adverse reactions, clinical characteristics, inborn errors of immunity, severe combined immunodeficiency

## Abstract

**Objectives:**

This study aimed to analyze the clinical characteristics and genetic features of children with adverse reactions to the Bacillus Calmette-Guérin (BCG) vaccine. The goal was to improve understanding of this condition, provide insights into early diagnosis and intervention, and support stratified management.

**Methods:**

Clinical data of 35 children hospitalized at Kunming Children’s Hospital between January 2014 and June 2024 with complete records and diagnosed with BCG vaccine adverse reactions were collected. Cases were classified into two groups: disseminated BCG disease (BCG-D) and BCG-itis. Children with primary immunodeficiency (PID) were further divided into severe combined immunodeficiency (SCID) and non-SCID groups. Clinical characteristics, immunological profiles, genetic backgrounds, and outcomes were compared between the groups.

**Results:**

Among the 35 cases, 25 were male, and 10 were female, with a median age of onset of 2 months (1–4 months). Eight cases (22.9%) were diagnosed with BCG-D, while 27 cases (77.1%) were classified as BCG-itis. Sixteen cases (45.7%) were confirmed to have PID, including SCID (7 cases, 20.0%), chronic granulomatous disease (6 cases, 17.1%), Mendelian susceptibility to mycobacterial disease (2 cases, 5.7%), and fas associated via death domain (FADD) gene mutation (1 case, 2.6%). Compared to the BCG-itis group, the BCG-D group exhibited significantly higher rates of fever, hepatosplenomegaly, elevated white blood cell counts, neutrophil counts, and C-reactive protein (CRP) levels, along with lower red blood cell counts and hemoglobin levels (p<0.05). Similarly, the SCID group showed significantly lower age, lymphocyte counts, IgM levels, CD3, CD4, and CD8 cell ratios, but higher CD19 cell ratios and mortality rates compared to the non-SCID group (p<0.05). Twenty-seven (77.1%) cases were discharged after improvement, and eight children (22.9%) succumbed to the condition, including six with SCID gene mutations (representing 85.7% of the total SCID cases), one with an interleukin 12 receptor subunit beta 1(IL12RB1) mutation, and one who was not genetically tested but diagnosed with disseminated BCG disease.

**Conclusions:**

In children presenting with adverse reactions to the BCG vaccine, the presence of fever, hepatosplenomegaly, elevated neutrophil levels, and CRP should prompt evaluation for disseminated BCG disease and assessment of immunological status. Early identification of underlying PID, particularly SCID, is crucial, given the high mortality and poor prognosis associated with the condition, necessitating timely interventions.

## Introduction

Tuberculosis (TB) remains a significant global public health issue. In 2014, the World Health Organization (WHO) introduced the “End TB Strategy,” one of whose pillars is the prevention of TB through vaccination of children under five years of age (Lienhardt et al., 2016). The Bacillus Calmette-Guérin (BCG) vaccine, developed by Albert Calmette and Camille Guérin in the early 20th century through the attenuation of live Mycobacterium bovis strains, is widely used to prevent miliary tuberculosis and tuberculous meningitis, especially in infants and young childre (Liao et al., 2022). BCG vaccination is utilized in 154 TB-endemic countries, including China, where the Shanghai D_2_PB302 strain is the standard. In China, BCG vaccination is mandatory for all newborns and is part of the routine immunization schedule. It is a single-dose regimen, administered at birth (within 24 to 48 hours). The vaccine is given via intradermal injection at a dose of 0.1 ml (Li et al., 2022). Following BCG vaccination, a localized immune response typically occurs, manifesting as induration, pustules, or scabbing at the injection site, which usually subsides within 12 weeks, leaving a scar. However, some individuals may experience localized or systemic adverse reactions. Local reactions include redness, abscess formation, or lymphadenitis at the injection site, while systemic reactions, such as osteomyelitis or disseminated BCG disease, may occur, with rapid disease progression and high mortality in some cases (Kourime et al., 2016; Nunes-Santos and Rosenzweig, 2018). Evidence suggests that individuals with underlying immunodeficiencies are at higher risk of both localized adverse reactions and disseminated infections, underscoring the need to evaluate immune status before vaccination (Al Busaidi et al., 2021).

Although several studies have reported on the adverse effects of the BCG vaccine, most research has focused on local reactions in immunocompetent children. There is a lack of comprehensive studies on the immunological and genetic factors contributing to adverse BCG reactions in children, especially those with underlying immune deficiencies. This gap in the literature highlights the importance of further investigation into the clinical characteristics, immunological profile, and genetic predispositions that may predispose certain individuals to severe vaccine reactions.

Moreover, early identification and management of children at risk of severe BCG-related adverse effects remain challenging. There is an urgent need for a better understanding of how to predict, diagnose, and intervene in such cases to reduce the risk of poor outcomes and improve patient survival.

This study collected data on 35 children with adverse reactions to the BCG vaccine, hospitalized at Kunming Children’s Hospital, to analyze their clinical characteristics, immunological features, genetic backgrounds, and outcomes. The aim was to enhance understanding of the condition, provide insights into diagnosis and intervention, and guide stratified management.

## Materials and methods

### Study population

This study collected clinical data on 35 children hospitalized at Kunming Children’s Hospital, a specialized pediatric hospital in Kunming, Yunnan Province, which serves as a tertiary referral center for pediatric infectious diseases, including complicated cases. These children were admitted between January 2014 and June 2024 with complete records and diagnosed with adverse reactions to the BCG vaccine. The 35 cases were exclusively from this hospital and do not represent the broader population of Yunnan Province. Ethical approval was obtained from the Ethics Review Committee of Kunming Medical University’s Children’s Hospital. Due to the retrospective design of the study, the requirement for informed consent was waived. All methods adhered to relevant guidelines and regulations, including the Declaration of Helsinki.

### Inclusion criteria

The diagnostic criteria for adverse reactions to the BCG vaccine were based on the Guidelines for Clinical Management of BCG Adverse Reactions issued by the Tuberculosis Division of the Chinese Medical Association (Lu, 2021):

BCG-itis diagnostic criteria:

1. History of BCG vaccination with local lymph node involvement (supraclavicular/cervical or ipsilateral axillary lymphadenitis, abscess, or sinus formation).
2. No history of contact with active TB patients.
3. Positive tuberculin skin test (TST) but negative interferon-gamma release assays (e.g., T-SPOT.TB or QuantiFERON-TB GOLD).
4. Acid-fast bacilli detected in lymph node aspirates or secretions; positive culture for Mycobacterium tuberculosis complex.
5. Histopathology of lymph nodes showing caseating necrosis and granuloma formation.
6. PCR amplification of lymph node aspirates or secretions indicating RD1 deletion or pncA deletion, confirming BCG infection.

A clinical diagnosis required meeting criteria 1, 2, 3, and either 4 or 5. A confirmed diagnosis required meeting criteria 1, 2, 3, and either 4 and 6 or 5 and 6.

Disseminated BCG disease diagnostic criteria:

1. Meeting the diagnostic criteria for BCG-itis.
2. Evidence of distant organ involvement (e.g., lungs, mediastinal or abdominal lymph nodes, skin, soft tissue, liver, spleen, kidneys, bones, testes, brain, or meninges) as indicated by imaging, histopathology, or microbiological findings, including acid-fast staining or culture.

### Exclusion criteria

1. HIV infection was ruled out in all children.
2. Incomplete clinical data.

### Grouping

Cases were classified based on the presence or absence of disseminated BCG infection into two groups: BCG-D and BCG-itis. Cases with inborn errors of immunity (IEI) were further divided into Severe Combined Immunodeficiency (SCID) and non-SCID groups, following the 2022 classification by the International Union of Immunological Societies (Tangye et al., 2022).

### laboratory procedures

Humoral Immunity Analysis: The levels of Immunoglobulin G (IgG), Immunoglobulin A (IgA), and Immunoglobulin M (IgM) were measured using nephelometry.

Flow cytometry was used to determine the percentage and absolute count of lymphocyte subsets. The methodology was as follows: Reagents and Equipment: The primary reagents included Multitest Reagent CD45-FITC/CD4-PE/CD8-ECD/CD3-PC5 and CD45-FITC/CD56-PE/CD19-ECD/CD3-PC5, along with hemolysis solution and flow-count fluorospheres (all purchased from Beckman Coulter, California, USA). The analysis was conducted using a Beckman Coulter NAVIOS flow cytometer.Sample Preparation and Detection: Peripheral blood samples were collected in ethylenediaminetetraacetic acid (EDTA) anticoagulation tubes via reverse sampling. A 50 μL aliquot of each sample was mixed with 20 μL of multi-antibody reagent, incubated at room temperature (20°C–25°C) in the dark for 15 minutes, followed by the addition of 2 mL of hemolysin for 10 minutes. Samples were then centrifuged at 1,500 r/min for 5 minutes (radius: 5 cm), and the supernatant was discarded. A 2 mL acid buffer solution was used for washing, followed by another centrifugation under the same conditions. After removing the supernatant, 500 μL of phosphate-buffered saline (PBS) and 50 μL of flow-count fluorospheres were added. Data from 15,000 cells were acquired using a Beckman Coulter NAVIOS instrument and analyzed with Kaluza Analysis Software version 2.1 (Beckman Coulter, California, USA). All experiments were performed in accordance with relevant guidelines and regulations.

Genetic Testing and Whole-Exome Sequencing (WES): Peripheral blood samples were sent to MyGenostics (Beijing, China) for WES. DNA was extracted using the Whole Blood DNA Extraction Kit (Qiagen, Hilden, Germany). The trimmed sequencing reads were mapped to the human reference genome (hg19). Variants were filtered using multiple databases, including dbSNP (https://www.ncbi.nlm.nih.gov/snp/), ClinVar (https://www.ncbi.nlm.nih.gov/clinvar/), the Genome Aggregation Database (gnomAD, http://gnomad.broadinstitute.org/), Exome Aggregation Consortium (ExAC, http://exac.broadinstitute.org/), 1000 Genomes Project (1000G, http://browser.1000genomes.org/), and the Human Gene Mutation Database (HGMD, http://www.hgmd.cf.ac.uk/). Mutations in CD3 Delta Subunit of T-Cell Receptor Complex (CD3D), Interleukin 2 Receptor Subunit Gamma (IL2RG), Cytochrome B-245 Beta Chain (CYBB), Fas-Associated via Death Domain (FADD), Interleukin 12 Receptor Subunit Beta 1 (IL12RB1), and Recombination Activating 2 (RAG2) genes were further validated by Sanger sequencing. Variant interpretation was conducted in accordance with the guidelines set by the American College of Medical Genetics and Genomics (ACMG).

### Data collection

Data collected included demographic information, clinical characteristics, laboratory findings (including blood tests, inflammatory markers, immunological indicators, and genetic results), and outcomes.

### Statistical analysis

Statistical analyses were performed using SPSS 26.0. Normally distributed continuous data were presented as mean ± standard deviation (x ± s) and compared using the t-test. Non-normally distributed data were expressed as median (Q1, Q3) and compared using the Mann-Whitney U test. Categorical variables were presented as numbers and percentages, with comparisons made using Fisher’s exact test or χ² test. A p-value of <0.05 (two-tailed) was considered statistically significant.

## Results

### General data of the children

**Figure 1:**
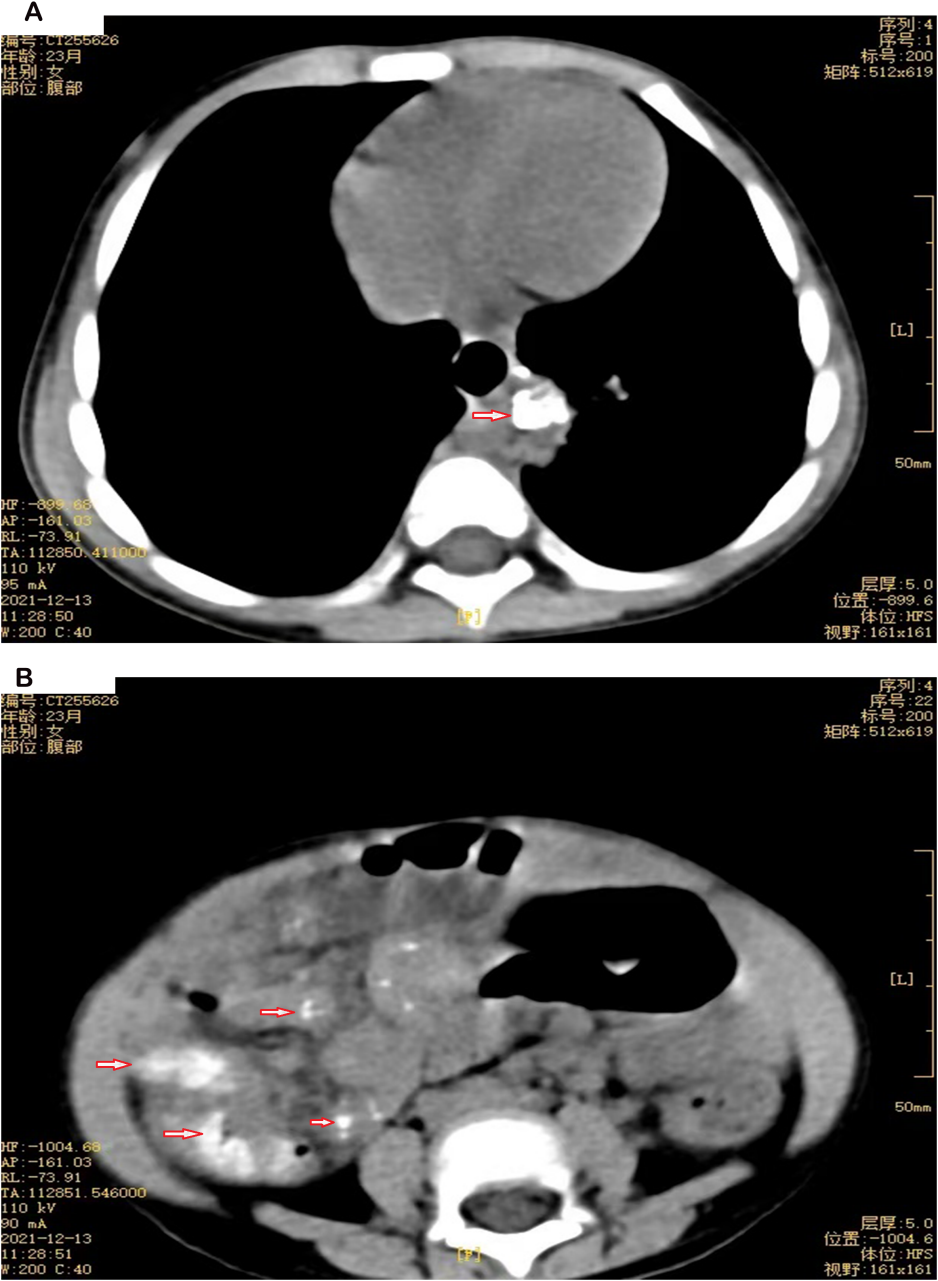
**(A)** Chest CT of P19, a 23-month-old female. The arrow points to soft tissue in the left posterior lower mediastinum with calcification. **(B)** Abdominal CT of P19, a 23-month-old female. The arrow indicates multiple nodules with calcification in the abdominal and mesenteric space.

A total of 35 children were included in the study, with 25 male and 10 female, yielding a male-to-female ratio of 2.5:1. The median age at onset was 2 months (range: 1–4 months), and the median age at diagnosis was 6 months (range: 4–9 months), with 27(77.1%) children diagnosed before 12 months of age. There were 8(22.6%) cases of disseminated BCG disease, including 4 cases with hematogenous spread, 1 case with intestinal infection (see **Figure 1**), 1 case with splenic lesions, and 2 cases with distant inguinal lymphadenopathy. The remaining 27(77.4%) cases were non-disseminated infections. Sixteen children (45.7%) were confirmed to have primary immunodeficiencies (PID). Detailed information is presented in **Table 1**.

**Table 1:** General Information, Clinical Manifestations, Underlying Diseases, and Prognosis of 35 Children with Adverse Reactions to Bacillus Calmette-Guérin (BCG) Vaccination.

ID	Gender	Diagnosis age, months	Onset age, months	Clinical manifestations	Disease group	Primary disease	Outcome
P1	Female	<1 year	<1 year	Fever, Left axillary lymphadenopathy, Pulmonary infection, Oral ulcerations, urinary tract infection	BCG-itis	Gene Testing Not Completed	Survived
P2	Male	<1 year	<1 year	Left axillary lymphadenopathy	BCG-itis	Gene Testing Not Completed	Survived
P3	Male	<1 year	<1 year	Left axillary lymphadenopathy, Thrombocytopenia	BCG-itis	Gene Testing Not Completed	Survived
P4	Male	<1 year	<1 year	Left axillary lymphadenopathy,	BCG-itis	Gene Testing Not Completed	Survived
P5	Male	<1 year	<1 year	Left axillary lymphadenopathy, Pulmonary infection, Perianal abscess	BCG-itis	Gene Testing Not Completed	Survived
P6	Male	<1 year	<1 year	Left axillary lymphadenopathy	BCG-itis	Gene Testing Not Completed	Survived
P7	Female	<1 year	<1 year	Left axillary lymphadenopathy, Malnutrition	BCG-itis	Gene Testing Not Completed	Survived
P8	Male	<1 year	<1 year	Left axillary lymphadenopathy, Hepatic injury	BCG-itis	Gene Testing Not Completed	Survived
P9	Male	<1 year	<1 year	Fever, Left axillary lymphadenopathy	BCG-itis	Gene Testing Not Completed	Survived
P10	Male	<1 year	<1 year	Left axillary lymphadenopathy, Malnutrition	BCG-itis	Gene Testing Not Completed	Survived
P11	Male	<1 year	<1 year	Left axillary lymphadenopathy, Malnutrition, Pulmonary infection	BCG-itis	Gene Testing Not Completed	Survived
P12	Female	<1 year	<1 year	Fever, Left axillary lymphadenopathy, Shock, Pulmonary infection, Hepatosplenomegaly, Anemia	BCG-D	Gene Testing Not Completed	Mortality
P13	Male	1–3 years	<1 year	Left axillary lymphadenopathy	BCG-itis	Gene Testing Not Completed	Survived
P14	Male	1–3 years	1–3 years	Left axillary lymphadenopathy	BCG-itis	Gene Testing Not Completed	Survived
P15	Male	1–3 years	1–3 years	Left axillary lymphadenopathy	BCG-itis	Gene Testing Not Completed	Survived
P16	Male	1–3 years	1–3 years	Left axillary lymphadenopathy	BCG-itis	Gene Testing Not Completed	Survived
P17	Female	<1 year	<1 year	Left axillary lymphadenopathy	BCG-itis	Gene Testing Not Completed	Survived
P18	Female	<1 year	<1 year	Fever, Left axillary lymphadenopathy, Malnutrition, Pulmonary infection, Dermatitis	BCG-itis	Completed Gene Testing, Negative Result	Survived
P19	Female	1–3 years	<1 year	Fever, Left axillary lymphadenopathy, Malnutrition, Pulmonary infection, Diarrhea, Inguinal lymphadenopathy,	BCG-D	Completed Gene Testing, Negative Result	Survived
P20	Female	<1 year	<1 year	Fever, Left axillary lymphadenopathy, Malnutrition, Pulmonary infection, Hepatosplenomegaly, Inguinal lymphadenopathy	BCG-D	CD3D(c.274+5G>A)hom	Mortality
P21	Male	<1 year	<1 year	Fever, Left axillary lymphadenopathy, Malnutrition, Pulmonary infection, Hepatosplenomegaly, Sepsis, Dermatitis, Purulent discharge from the ear canal	BCG-D	IL2RG:c.982C>T (p.R328X)	Mortality
P22	Male	<1 year	<1 year	Fever, Left axillary lymphadenopathy, Pulmonary infection	BCG-itis	CYBB: c.388C>T (p.R130X)	Survived
P23	Female	<1 year	<1 year	Left axillary lymphadenopathy, Hepatosplenomegaly, Diarrhea	BCG-itis	FADD:c.350G>A (p.R117H) hom	Survived
P24	Male	<1 year	<1 year	Fever, Left axillary lymphadenopathy, Malnutrition, Pulmonary infection, Hepatosplenomegaly	BCG-itis	IL2RG:c.664C>T (p.R222C)	Mortality
P25	Female	<1 year	<1 year	Fever, Left axillary lymphadenopathy, Inguinal lymphadenopathy, Hepatosplenomegaly, Sepsis	BCG-D	IL12RB1 c.635G>A (p.R212Q) hom	Survived
P26	Male	1–3 years	<1 year	Fever, Left axillary lymphadenopathy, Malnutrition, Hepatomegaly, Perianal abscess	BCG-itis	CYBB:c.46-8_85del	Survived
P27	Male	<1 year	<1 year	Fever, Left axillary lymphadenopathy, Malnutrition, Hepatomegaly, Pulmonary infection, Diarrhea, Oral thrush	BCG-itis	RAG2: c.668C>A (p.S223X) hom	Mortality
P28	Male	<1 year	<1 year	Left axillary lymphadenopathy, Malnutrition, Pulmonary infection, Diarrhea, Oral thrush	BCG-itis	RAG2: c.668C>A (p.S223X) hom	Mortality
P29	Male	<1 year	<1 year	Fever, Left axillary lymphadenopathy, Hepatomegaly, Pulmonary infection, Pneumocystis jirovecii infection	BCG-itis	DCLRE1C: Exon 1-3 Deletion	Mortality
P30	Female	<1 year	<1 year	Fever, Left axillary lymphadenopathy, Hepatosplenomegaly, Pulmonary infection	BCG-D	IL12RB1: c.1148G>A (p.C383Y) hom	Mortality
P31	Male	1–3 years	<1 year	Fever, Left axillary lymphadenopathy, Pulmonary infection	BCG-itis	CYBB Gene Mutation	Survived
P32	Male	<1 year	<1 year	Fever, Left axillary lymphadenopathy, Malnutrition, Hepatosplenomegaly, Pulmonary infection, Shock, Pneumocystis jirovecii infection, CMV infection	BCG-D	RAG2:c.1275_1278dup (p. I427Gfs12),c.413A>G(p.Y138C)	Survived
P33	Male	<1 year	<1 year	Fever, Left axillary lymphadenopathy, Pulmonary infection, Inguinal lymphadenopathy, purulent secretion	BCG-D	CYBB:c.481A>T(p.K161X)	Survived
P34	Male	<1 year	<1 year	Fever, Left axillary lymphadenopathy, Malnutrition, Pulmonary infection	BCG-itis	CYBB:c.742dup(p.I248Nfs36)hom	Survived
P35	Male	1–3 years	NA	Fever, Left axillary lymphadenopathy, Pulmonary infection	BCG-itis	CYBB Gene Mutation	Survived

CD3D, CD3 delta subunit of T-cell receptor complex; IL2RG, interleukin 2 receptor subunit gamma; CYBB, cytochrome b-245 beta chain; FADD, Fas associated via death domain; IL12RB1; interleukin 12 receptor subunit beta 1; RAG2, recombination activating 2.

### Comparison between disseminated BCG disease and BCG-ITIS

Compared to the BCG-itis group, the BCG-D group exhibited significantly higher rates of fever, hepatosplenomegaly, elevated white blood cell counts, neutrophil counts, and C-reactive protein (CRP) levels. Additionally, the BCG-D group had lower red blood cell counts and hemoglobin levels (p<0.05). Specific data are presented in **Table 2**.

**Table 2:** Comparison of Clinical Features and Laboratory Results between the BCG-D Group and the BCG-itis Group.

Characteristics	BCG-D (n=8)	BCG-itis (n=27)	p-value
Basic Information			
Age at Diagnosis, Median (Q1, Q3), months	6.50 (5.25, 8.00)	5.00 (4.00, 13.00)	0.552
Age at Onset, Median (Q1, Q3), months	2.00(1.25,2.75)	2.00(1.00,4.00)	0.429
Gender, Male, n (%)	3 (37.5%)	22 (81.5%)	0.027
Fever, n (%)	7 (87.5%)	11 (40.7%)	0.041
Malnutrition, n (%)	5 (62.5%)	10 (37%)	0.246
Hepatomegaly, n (%)	5 (62.5%)	5 (18.5%)	0.027
Splenomegaly, n (%)	5 (62.5%)	2 (7.4%)	0.03
Pulmonary Infection, n (%)	7 (87.5%)	14 (51.9%)	0.108
Laboratory Examination			
White Blood Cell Count, Median (Q1, Q3) ,10^9^/L	22.50 (16.91, 26.66)	10.47 (7.06, 16.68)	0.001
Neutrophil Count, Median (Q1, Q3) ,10^9^/ L	12.78 (9.46, 19.24)	3.06 (1.43, 5.08)	0.001
Lymphocyte Count, Median (Q1, Q3) ,10^9^/ L	4.76 (2.87, 8.67)	5.9 (3.36, 6.90)	0.844
Red Blood Cell Count, Median (Q1, Q3) ,10^12^/ L	3.93 (2.70, 4.40)	4.63 (4.47, 4.95)	0.001
Hemoglobin, Median (Q1, Q3), g/L	98.50 (64.75, 112.25)	120.00 (115.00, 126.00)	0.002
Platelet Count, Median (Q1, Q3) ,10^9^/L	354.00 (122.75, 525.25)	362.00 (273.00, 483.00)	0.709
CRP, Median (Q1, Q3), mg/L	44.34 (4.73, 79.96)	1.78 (0.50, 23.94)	0.028
IgG, Median (Q1, Q3), g/L	9.70 (1.00, 13.29)	5.57 (4.81, 7.11)	0.364
IgM, Median (Q1, Q3), g/L	0.87 (0.20, 1.22)	0.68 (0.33, 1.42)	0.801
IgA, Median (Q1, Q3), g/L	0.55 (0.07, 0.89)	0.14 (0.06, 0.39)	0.129
CD3 Ratio, Median (Q1, Q3), %	39.27 (28.34, 54.44)	58.32 (38.53, 65.72)	0.184
CD4 Ratio, Median (Q1, Q3), %	26.62 (10.34, 34.46)	31.37 (12.88, 39.33)	0.658
CD8 Ratio, Median (Q1, Q3),%	10.81 (3.40, 16.76)	18.88 (15.32, 22.45)	0.06
CD19 Ratio, Median (Q1, Q3), %	35.57 (7.22, 60.59)	20.54 (15.82, 31.98)	0.109
CD56 Ratio, Median (Q1, Q3),%	9.94 (7.23, 23.99)	15.35 (10.20, 25.38)	0.439
Outcome			
Mortality, n (%)	3 (37.5%)	4 (14.8%)	0.312

### Comparison between SCID and non-SCID groups

In total, 16 cases (45.7%) of IEI were diagnosed, including 7 cases of SCID (43.75%) with gene mutations in CD3D (1 case), DCLRE1C (1 case), IL2RG (2 cases), and RAG2 (3 cases). Six cases (37.5%) had chronic granulomatous disease (CGD) due to CYBB gene mutations, 2 cases (12.5%) had Mendelian susceptibility to mycobacterial disease (MSMD) due to IL12RB1 mutations, and 1 case (6.25%) had a FADD gene mutation. The cases were derived from 15 families (P27 and P28 were siblings), and 4 cases had consanguineous marriages (P23, P25, P27, P28). Two cases had clear family histories, such as unexplained neonatal deaths (P26 and P33). Four cases were from ethnic minorities (Dai ethnicity (P21), Tibetan ethnicity (P23), Yi ethnicity (P26), and Hani ethnicity (P33)). Among the 16 IEI cases, 12 were male and 4 were female, all presenting with onset before 6 months of age. In the 16 IEI cases, 6 had disseminated infections, 13 had fever, 10 had malnutrition, 9 had hepatomegaly, 6 had splenomegaly, 13 had pulmonary infections, and 4 had enlarged lymph nodes at non-vaccination sites. The SCID group showed significantly lower age at onset, lymphocyte counts, IgM levels, and CD3, CD4, and CD8 cell ratios, but higher CD19 cell ratios and mortality rates compared to the non-SCID group (p<0.05). Detailed data are presented in **Table 3**.

**Table 3:** Comparison of Clinical Features and Laboratory Results between the SCID Group and the Non-SCID Group.

Characteristics	Non-SCID Group (n=9)	SCID Group (n=7)	p-value
Basic Information			
Age at Diagnosis, Median (Q1, Q3), months	8.00 (6.00, 16.00)	5.00 (4.00, 6.00)	0.009
Age at Onset, Median (Q1, Q3), months	1.00 (1.00, 4.00)	1.00 (1.00, 2.00)	0.909
Gender, Male, n (%)	6 (66.7%)	6 (85.7%)	0.585
Fever, n (%)	7 (77.8%)	6 (85.7%)	1
Malnutrition, n (%)	4 (44.4%)	6 (85.7%)	0.145
Hepatomegaly, n (%)	3 (33.3%)	6 (85.7%)	0.06
Splenomegaly, n (%)	2 (22.2%)	4 (57.1%)	0.302
Pulmonary Infection, n (%)	6 (66.7%)	7 (100%)	0.213
Laboratory Examination			
White Blood Cell Count, Median (Q1,Q3) ,10^9^/L	20.59 (16.03, 25.26)	16.49 (3.25, 23.36)	0.186
Neutrophil Count, Median (Q1, Q3) ,10^9^/ L	10.31 (4.21, 13.37)	5.89 (1.43, 13.03)	0.56
Lymphocyte Count, Median (Q1, Q3) ,10^9^/ L	8.84 (5.27, 11.28)	2.31 (0.65, 6.26)	0.013
Red Blood Cell Count, Median (Q1, Q3) ,10^12^/ L	4.42 (4.02, 4.86)	4.57 (4.35, 4.79)	1
Hemoglobin, Median (Q1, Q3), g/L	107 (97.5, 120.5)	114 (107, 116)	0.596
Platelet Count, Median (Q1, Q3) ,10^9^/L	390 (164, 496.5)	327 (213, 531)	0.832
CRP, Median (Q1, Q3), mg/L	46.46 (12.8, 97.65)	13.65 (2.11, 50.59)	0.315
IgG, Median (Q1, Q3), g/L	8.92 (6.68, 10.75)	0.23 (0.13, 7.145)	0.053
IgM, Median (Q1, Q3), g/L	0.80 (0.6, 1.625)	0.01 (0.01, 0.02)	0.007
IgA, Median (Q1, Q3), g/L	0.64 (0.24, 0.9)	0.01 (0.01, 0.01)	0.005
CD3 Ratio, Median (Q1, Q3), %	53.23 (40.16, 69.64)	4.16 (1.06, 28.34)	0.011
CD4 Ratio, Median (Q1, Q3), %	26.31 (17.46, 34.31)	0.82 (0.16, 10.34)	0.021
CD8 Ratio, Median (Q1, Q3), %	17.21 (16.71, 21.16)	2.69 (0.71, 8.62)	0.002
CD19 Ratio, Median (Q1, Q3), %	35.08 (20.85, 36.83)	0.45 (0.35, 33.97)	0.049
CD56 Ratio, Median (Q1, Q3), %	9.80 (6.66, 20.48)	88.81 (19.92, 94.42)	0.021
Outcome			
Mortality, n (%)	1 (11.1%)	6 (85.7%)	0.009

### Treatment and follow-up

In cases diagnosed with BCG-itis, if lymph node abscesses failed to resolve after 6–9 months of observation and were ≥3 cm in diameter, or if simple lymphadenitis progressed to suppurative forms, needle aspiration of the abscess was performed, and the prognosis was generally good. For cases diagnosed with disseminated BCG disease, a 4-drug regimen of isoniazid, rifampin, ethambutol, and linezolid was administered for anti-tuberculosis treatment. During follow-up, 8 children (22.8%) died, including 6 with SCID-related gene mutations (88.5% of the total SCID cases), 1 with an IL12RB1 gene mutation, and 1 case who was not genetically tested but diagnosed with disseminated BCG disease. Among the eight deceased patients, the primary causes of death included septic shock (P12, P20), multiple organ failure due to infections (P21, P30), severe pneumonia and acute respiratory distress syndrome (ARDS) (P24, P27, P29), and severe gastrointestinal infections (P28).

## Discussion

BCG is a widely used live attenuated vaccine that has been in use for a long time and has proven effective in protecting children under five years of age from severe TB (Principi and Esposito, 2015). For most children, BCG vaccination is safe, but adverse reactions, such as BCG-related lymphadenitis and disseminated BCG disease, can occur (Sohani et al., 2020). The incidence of these reactions ranges from 1 in 10,000 to 1 in 1,000,000, with clinical manifestations, complications, and prognosis differing according to underlying conditions (Engelis et al., 2016). This study aimed to retrospectively analyze the clinical characteristics and genetic background of children with BCG vaccine-related adverse reactions to enable early intervention and minimize the damage from the disease and medical resource waste.

In this study, 35 children with BCG-related adverse reactions were included, all of whom exhibited BCG-reactive lymphadenitis, mainly in the left axillary lymph nodes, typically occurring within six months after vaccination. No significant difference was found in the age of onset or diagnostic age between the groups. However, children in the BCG-D group exhibited a higher proportion of fever and hepatosplenomegaly, as well as elevated white blood cell counts, neutrophil counts, and C-reactive protein levels. These findings suggest that, in addition to reactive lymphadenitis, disseminated BCG infection leads to more severe systemic manifestations due to the dissemination of the vaccine, indicating a more intense inflammatory response (Rezai et al., 2008; Marouli et al., 2023).

In patients with IEI, the incidence and severity of infectious diseases often exceed normal levels, and infections caused by opportunistic pathogens or rare microorganisms are more likely to occur (Tangye et al., 2022). Casanova et al (Casanova et al., 1995). reviewed 121 reported cases of disseminated BCG infection and found that 61 cases had identifiable immune deficiencies, with 45 cases of SCID, 11 cases of CGD, 4 cases of acquired immunodeficiency syndrome, and 1 case of complete DiGeorge syndrome (CDGS). Our study confirmed 16 cases of IEI, including 7 cases of SCID (43.75%), 6 cases of CGD (37.5%), 2 cases of MSMD (12.5%), and 1 case of FADD gene mutation (6.25%). The predominance of SCID in our study was consistent with the literature, although the proportions differed slightly from a report from the pediatric hospital at Fudan University, which classified 62.5% as CGD, 25% as SCID, and 12.5% as MSMD (Zeng et al., 2023). Among the 16 IEI patients, 6 developed disseminated BCG disease, suggesting that children with underlying immune deficiencies are at a higher risk of severe adverse reactions, particularly disseminated BCG disease. This finding is consistent with previous reports emphasizing the correlation between immune system dysfunction and increased susceptibility to severe vaccine-related complications (Norouzi et al., 2012).One study emphasized that disseminated BCG disease is particularly common in individuals with congenital immune deficiencies. This suggests that screening and careful assessment may be required before administering live vaccines to this vulnerable group (Yadav et al., 2021). The results of our study reinforce the importance of early identification of high-risk children, which could improve patient prognosis.

SCID is the most severe form of IEI, characterized by abnormal development and functional defects of T lymphocytes, which predisposes patients to a wide range of infections, including those caused by low-virulence pathogens like BCG (Dvorak et al., 2023). SCID is considered a pediatric emergency. Low white blood cell counts and lymphocyte counts are hallmark features of SCID (Burns et al., 2021). When comparing SCID with non-SCID groups, the SCID group exhibited significantly lower lymphocyte counts, CD3, CD4, and CD8 cell ratios, and a higher mortality rate. These findings highlight the importance of early identification of SCID (Wang et al., 2021). Although lymphocyte count is a key indicator of SCID, it has low sensitivity and specificity. In 2010, the United States introduced the screening of T cell receptor excision circles (TREC), a biomarker of T cell precursors, for T cell deficiency diseases. TREC screening has high sensitivity and specificity for identifying SCID and clinically significant T cell deficiencies (Amatuni et al., 2019; Puck, 2019). These findings underscore the need for tailored vaccination recommendations for children with immunodeficiency to minimize associated risks (Sellami et al., 2018).

Although our study provides some insights into the clinical diagnosis and treatment of BCG vaccine-related adverse reactions, several limitations must be acknowledged. First,the relatively small sample size limits the generalizability of our findings, and future studies should aim to increase the sample size to validate our results. Furthermore, this study is a retrospective analysis, which inherently carries a potential risk of selection bias. Additionally, incomplete clinical data for some pediatric patients may have influenced the conclusions drawn. Lastly, a comprehensive genomic analysis was not conducted for all cases in this study, which may have resulted in the omission of certain potential genetic mutations or immunodeficiencies, warranting further exploration in future research.

## Conclusion

In children exhibiting adverse reactions to the BCG vaccine, the occurrence of fever, hepatosplenomegaly, elevated neutrophil counts, and increased CRP levels should raise suspicion for disseminated BCG disease and warrant an evaluation of their immunological status. Early recognition of underlying PID, especially SCID, is essential due to its high mortality rate and unfavorable prognosis, underscoring the need for prompt intervention.

## Data Availability

The raw data supporting the conclusions of this article will be made available by the authors, without undue reservation.
